# Immune Reconstitution After Autologous Hematopoietic Stem Cell Transplantation in Crohn’s Disease: Current Status and Future Directions. A Review on Behalf of the EBMT Autoimmune Diseases Working Party and the Autologous Stem Cell Transplantation In Refractory CD—Low Intensity Therapy Evaluation Study Investigators

**DOI:** 10.3389/fimmu.2018.00646

**Published:** 2018-04-04

**Authors:** Alan Graham Pockley, James O. Lindsay, Gemma A. Foulds, Sergio Rutella, John G. Gribben, Tobias Alexander, John A. Snowden

**Affiliations:** ^1^John van Geest Cancer Research Centre, School of Science and Technology, Nottingham Trent University, Nottingham, United Kingdom; ^2^Centre for Immunobiology, Barts and the London School of Medicine and Dentistry, Blizard Institute, Queen Mary University of London, London, United Kingdom; ^3^Centre for Haemato-Oncology, Barts Cancer Institute, Queen Mary University of London, London, United Kingdom; ^4^Department of Rheumatology and Clinical Immunology, Charité – University Medicine, Berlin, Germany; ^5^German Rheumatism Research Center Berlin (DRFZ) – a Leibniz Institute, Berlin, Germany; ^6^Department of Haematology, Sheffield Teaching Hospitals NHS Foundation Trust, Sheffield, United Kingdom

**Keywords:** autologous stem cell transplantation, Crohn’s disease, hematopoietic stem cell transplantation, immune reconstitution, inflammatory bowel diseases, T cell receptor repertoire

## Abstract

Patients with treatment refractory Crohn’s disease (CD) suffer debilitating symptoms, poor quality of life, and reduced work productivity. Surgery to resect inflamed and fibrotic intestine may mandate creation of a stoma and is often declined by patients. Such patients continue to be exposed to medical therapy that is ineffective, often expensive and still associated with a burden of adverse effects. Over the last two decades, autologous hematopoietic stem cell transplantation (auto-HSCT) has emerged as a promising treatment option for patients with severe autoimmune diseases (ADs). Mechanistic studies have provided proof of concept that auto-HSCT can restore immunological tolerance in chronic autoimmunity *via* the eradication of pathological immune responses and a profound reconfiguration of the immune system. Herein, we review current experience of auto-HSCT for the treatment of CD as well as approaches that have been used to monitor immune reconstitution following auto-HSCT in patients with ADs, including CD. We also detail immune reconstitution studies that have been integrated into the randomized controlled Autologous Stem cell Transplantation In refractory CD—Low Intensity Therapy Evaluation trial, which is designed to test the hypothesis that auto-HSCT using reduced intensity mobilization and conditioning regimens will be a safe and effective means of inducing sustained control in refractory CD compared to standard of care. Immunological profiling will generate insight into the pathogenesis of the disease, restoration of responsiveness to anti-TNF therapy in patients with recurrence of endoscopic disease and immunological events that precede the onset of disease in patients that relapse after auto-HSCT.

## Introduction

### Etiology, Epidemiology, and Management of Crohn’s Disease (CD)

The intestinal inflammation associated with CD is caused by mucosal immune system reactivity to luminal antigen in genetically susceptible individuals. Active intestinal inflammation is associated with dysbiosis of the fecal and mucosal microbiota and increased intestinal permeability with alterations in innate lymphoid cell (ILC) populations. Defects in the innate immune pathway in CD are implicated by pathogenic mutations in the nucleotide-binding oligomerization domain 2 (*NOD2*) and autophagy-related protein 16-1 (*ATG16L1)* genes which suggest impairment of bacterial sensing and clearance. CD is associated with increased IL-12/IL-23 release from antigen-presenting cells and an imbalance in the differentiation of Th1/Th17 effector and regulatory lymphocytes (1, 2).

The incidence of CD is increasing in young adults who live with their disease for six decades (3). In addition, there has been a significant increase in CD incidence in newly industrialized countries of Africa, Asia, and South America (4). The prevalence of CD in the UK stands at approximately 145/100,000 (5) with 9.5 new cases per 100,000 annually (6). The highest prevalence in North America is reported in Canada at 319/100,000 (4). Traditional medical management focuses on controlling intestinal inflammation using conventional or biological therapy. Although many patients respond to first line biologic therapy, a recent prospective UK registry of 1,500 patients with CD commencing their first anti-TNF therapy reports primary non-response in between 16.9 and 23.7% of patients (7) and a secondary loss of response in a further 29% of patients over 2 years (7).

Recent single and multicenter cohort studies have reported a significant burden of adverse events with anti-TNF therapies including an increased risk of lymphoma independent of the use of concomitant thiopurine therapy (8, 9). The anti-integrin vedolizumab and the IL-12/23 antibody ustekinumab are both licensed as second-line biologic therapy for refractory CD. However, both therapies are less effective at inducing and maintaining remission in patients that have been exposed to anti-TNF therapy previously (10). Although novel biological therapies are in clinical development none have reported short- or long-term remission rates in more than 50% patients. Furthermore, biologic therapies currently constitute the largest proportion of the total treatment costs of patients with CD (11).

Patients refractory to medical therapy and those who develop stricturing or penetrating complications of disease progression require surgical resection of the affected intestine (12). However, disease recurrence after surgery is common and many patients face repeated or extensive surgeries that may require a stoma or result in short bowel syndrome and a requirement for parenteral nutrition support. Although the introduction of biologic therapies has been associated with a reduction in the rates of surgery over the recent decade, there has been no reduction in the requirement for repeat surgery (13). Patients with active disease refractory to currently licensed therapies in whom surgery is inappropriate or declined face ineffective biologic therapy and frequent courses of corticosteroids which are associated with a significant burden of treatment-related morbidity and mortality and high health-care resource utilization (11). There is a clear unmet need for an effective long-term therapy for this cohort of patients.

### Clinical Studies Investigating Auto-HSCT for CD

Over the last two decades, autologous hematopoietic stem cell transplantation (auto-HSCT) has been identified as a promising therapeutic option for patients with severe autoimmune diseases (ADs). Mechanistic studies suggest that restoration of immunological tolerance in chronic autoimmunity occurs after auto-HSCT *via* eradication of immune memory and reconfiguration of the immune system. Although case reports suggested exceptional benefit for patients with refractory CD after auto-HSCT (14, 15), concerns about safety and a lack of understanding as to whether benefit relates to the chemotherapeutic agents administered during mobilization/pre-transplant conditioning regimen or the transplant itself, led to the Autologous Stem cell Transplantation International Crohn’s disease (ASTIC) randomized controlled trial (NCT00297193) (16, 17). This was conducted at 11 accredited centers in 6 European countries (16, 17).

Autologous Stem cell Transplantation International Crohn’s disease compared cyclophosphamide mobilization alone to mobilization, high-dose chemotherapy, and auto-HSCT in patients with refractory CD (16, 17). Eligible patients underwent peripheral blood stem cell mobilization with high-dose cyclophosphamide (4 g/m^2^) and granulocyte colony-stimulating factor (G-CSF), after which they were randomized to immediate auto-HSCT or conventional care for 1 year (16, 17).

The primary endpoint for the trial was defined as clinical disease remission (CDAI < 150) for 3 months, with no medication for CD and no evidence of active disease on imaging and endoscopy at 1 year. Few patients randomized to immediate HSCT or who underwent mobilization and were then randomized to conventional care achieved the ambitious primary endpoint at 1 year. However, ASTIC did demonstrate benefits of auto-HSCT over conventional care in more traditional endpoints for therapeutic trials in this area (18), such as steroid-free clinical remission (CR) and mucosal healing (16). In addition, after the primary endpoint had been assessed, patients who had undergone mobilization and then been randomized to conventional care were offered auto-HSCT and then followed for a further year with identical assessments as in the randomized trial. Subsequent analysis of the 38 patients who underwent auto-HSCT in the ASTIC program and had data at baseline and 1 year reported a significant reduction in clinical and endoscopic disease activity at 1 year, with 19 out of 38 (50%) patients showing regression of all endoscopic ulceration (17). There were also significant improvements in quality of life between baseline and 1 year after auto-HSCT (17, 19, 20). Importantly, disease recurrence after HSCT responded to the introduction of anti-TNF therapies (15, 17). The doses of cyclophosphamide used in both groups resulted in significant numbers of adverse events and one death (17, 21, 22). Subsequent expert review has suggested that the high dose cyclophosphamide regimen used at both mobilization and conditioning was a factor for many of the adverse events (17, 21, 22). This view is supported by the outcome of an uncontrolled series of 14 patients with refractory Crohn’s disease (CD) who underwent auto-HSCT using a lower dose of cyclophosphamide during mobilization (2 g/m^2^) and conditioning (50 mg/kg for 4 days). The median duration of anemia and neutropenia was shorter after both mobilization and conditioning than that seen in previous reports using higher cyclophosphamide dosing, and few episodes of febrile neutropenia were reported. The lower intensity regimen still resulted in marked reduction in clinical disease activity with 13 patients achieving disease remission (CDAI < 150) at 30 days (20).

In addition, favorable long-term outcome after auto-HSCT in a single-center cohort of 29 patients with CD (some of whom participated in the ASTIC trial) has been described (23). This includes 5-year follow-up data with scheduled clinical, endoscopic and radiological assessment. Drug-free clinical and endoscopic remission (CDAI < 150, SES-CD < 7) was seen in 61% at 1 year, 52% at 2 years, 47% at 3 years, 39% at 4 years, and 15% at 5 years. However, 80% of those patients who experienced a relapse responded to the re-introduction of anti-TNF therapy. Six out of the 29 underwent surgery after auto-HSCT, and 1 patient died of CMV infection.

### Current Data on HSCT for CD From the EBMT Autoimmune Disease Working Party (ADWP) Registry

The long-term outcomes for all adult patients undergoing auto-HSCT for CD in Europe between 1997 and 2015 (outside the ASTIC study) have been evaluated using the EBMT registry. Clinical data were obtained for 82 patients from 19 centers in 7 countries, with clinical response being categorized as remission (no abdominal pain and normal stool frequency), significant improvement (improved pain and frequency), no change, or worsening of symptoms.

Median follow-up was 41 months (range 6–174). At 100 days post-HSCT, 64% of patients were in CR and a further 28% had experienced significant improvement. At 1 year after transplantation, data from 75 patients indicated that 43% were in CR, 20% improved, 17% unchanged and 20% worsened. 37% patients required surgery after auto-HSCT, and 73% re-started medical therapy. Of those requiring further treatment, 57% responded to therapies to which they had previously been refractory. Treatment-free survival, defined as survival without major surgery or medical therapy, was 27 and 22% at 3 and 5 years respectively.

In addition, the EBMT ADWP has produced a historical summary of the AD section of the registry, which has characterized outcomes following auto-HSCT in various indications. Compared with MS, relapse is more common in CD, which may reflect fundamental differences in disease processes (24).

**Figure 1:**
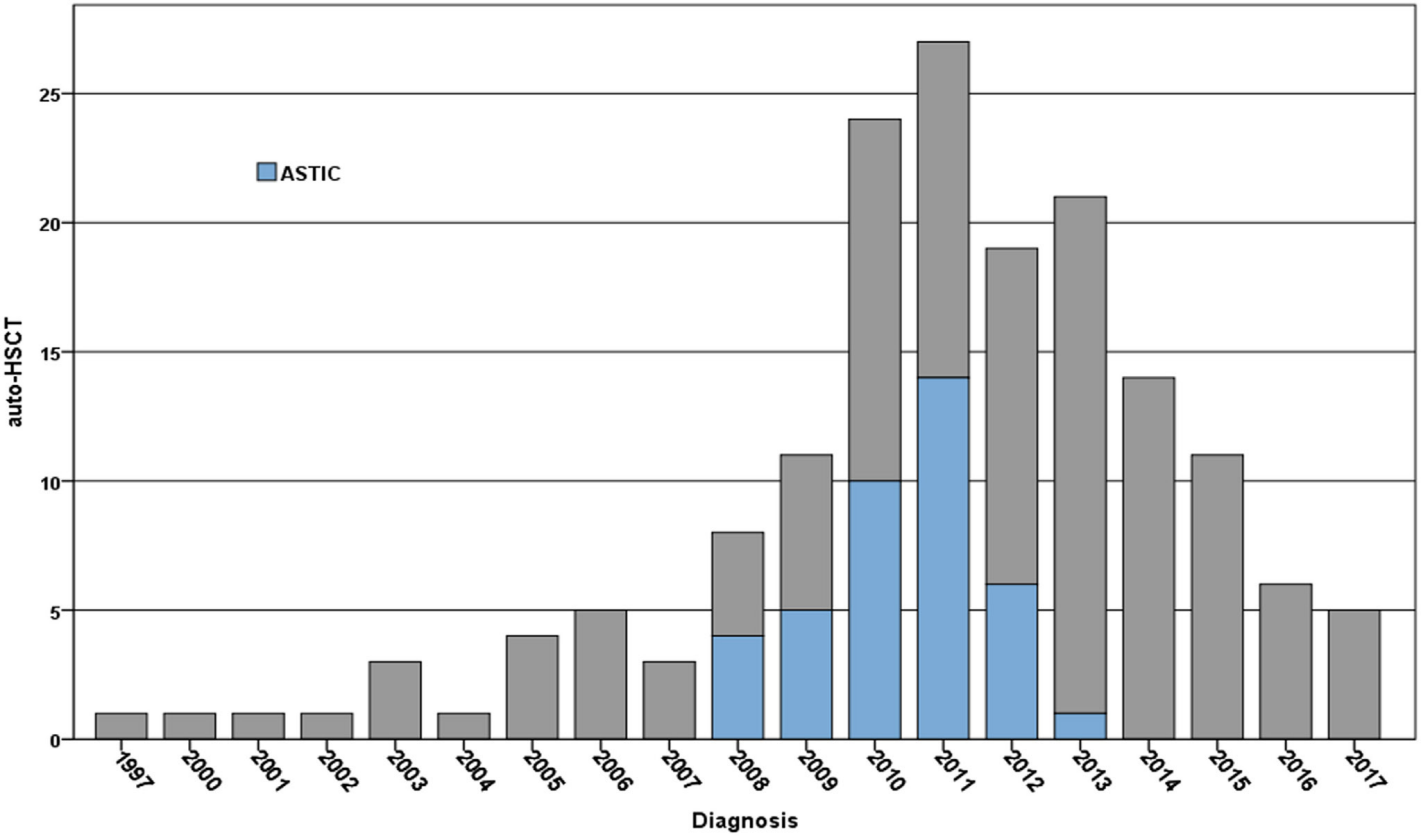
Registrations per year for the treatment of Crohn’s disease using autologous hematopoietic stem cell transplantation (auto-HSCT) within the EBMT registry 1994–2017 (note data for 2017 up to November 2017)—numbers treated on the Autologous Stem cell Transplantation International Crohn’s disease (ASTIC) trial are indicated in blue. Patients have been predominantly treated in Spain, UK, Italy, Belgium, The Netherlands, and France in reducing order (source EBMT Office, Paris).

As of November 2017, there have been a total of 166 transplant registrations for auto-HSCT of CD within the EBMT registry, with the majority 91.5% being in adults over 18 and 24% being treated as part of the ASTIC trial. Patients are predominantly being treated in Spain, UK, Italy, Belgium, The Netherlands, and France (Figure 1) (source, EBMT Office, Paris).

Taken together, current experience indicates that auto-HSCT can induce clinical and endoscopic remission, but does not result in sustained treatment-free disease remission in most patients. However, many patients become responsive to treatments to which they were previously refractory. Biomarkers that predict which patients will respond to anti-TNF therapy after auto-HSCT have not been identified and should be a focus of future trials in this area. In addition, auto-HSCT regimens including high dose cyclophosphamide are associated with significant adverse events, which may be avoided with lower intensity regiments. Hence further randomized controlled trials that assess the benefit of lower intensity regimens are warranted.

## Immune Reconstitution Following Auto-HSCT for CD

Specific studies of immune reconstitution in CD patients after auto-HSCT are limited to a few observations (25, 26). Therefore, this review focuses on the general literature relating to immune reconstitution in ADs and highlights how it might be best investigated in patients with CD to identify mechanisms of action of auto-HSCT and gain insights into the pathogenesis of CD.

### Rationale and Mechanistic Studies of Auto-HSCT for ADs

The original goal of auto-HSCT in ADs was to eradicate existing autoreactive immunological memory and regenerate a naïve, self-tolerant immune system (27, 28). Auto-HSCT has been shown to profoundly impact the immune system, as indicated by the regeneration of naïve B cells (29, 30), thymic reactivation (29, 31–33), the emergence of a polyclonal T cell receptor (TCR) repertoire (29, 31, 33), and restoration of Foxp3^+^ regulatory T (Treg) (34, 35) and B regulatory cell levels (36). In addition, disease associated restriction of the peripheral blood Treg TCR repertoire is completely reversed by auto-HSCT, both in terms of number and uniqueness of Treg cell TCR sequences (37). Functional assays investigating the fate of autoreactive T cells post-transplantation are limited due to restricted access to such cell clones in human disease compared to animal models. Nevertheless, *in vitro* stimulation assays in SLE have been able to demonstrate that T cell responses to antinuclear antigens were abolished, whereas virus-specific T cells were readily detectable in the first months after HSCT (29). Likewise, stimulation assays with myelin oligodendrocyte glycoprotein peptides in auto-HSCT-treated MS patients revealed reduced interleukin-17 responses and enhanced transforming growth factor-β1 production compared to natalizumab-treated patients, suggesting removal of autoreactive T cell clones as well as enhanced suppressive responses after HSCT (38). Collectively, these observations suggest that auto-HSCT has indeed the potential to restore self-tolerance by “resetting” the chronic autoreactive immune system into a naïve state.

Compared with MS and rheumatological diseases, there are relatively little data specifically relating to immune reconstitution following auto-HSCT in CD. The following sections will discuss how components of aberrant immunity may be investigated further in CD.

### Impact of Auto-HSCT for CD on the Innate Immune System

The intestinal innate immune system not only protects against invading pathogens, it also regulates the interactions between the commensal intestinal microbiota and the host at multiple levels, many of which are altered in CD. A small study investigating the effect of auto-HSCT on the innate immune system in seven patients with moderate to severe CD has reported elevated numbers of peripheral blood TLR-4 expressing monocytes that also express TNF-alpha and IFN-gamma at baseline compared to healthy controls, which were normalized after transplantation (25). The impact of auto-HSCT on other intestinal innate immune populations has not been assessed. Studies on ILCs would be of particular relevance given their role in orchestrating immune defense and regulation at mucosal surfaces and their role in CD pathogenesis.

### Impact of Auto-HSCT for CD on the Adaptive Immune System

The increased responses of CD4^+^ Th17/Th1 cells toward bacterial-derived proteins that have been described in patients with CD suggest a potential mechanism for sustaining persistent disease (39). Elimination of such cells *in vivo* by immunoablative chemotherapy is presumed to ameliorate intestinal inflammation. Conversely, recurrence of regulatory Foxp3^+^ Treg cells could restore self-regulation. The first investigation of the impact of auto-HSCT on peripheral blood lymphocytes in patients with CD demonstrated restoration of dysregulated T effector cell responses with a normalization in the number of IFN-gamma-secreting CD4^+^ T cells together with a significant increase in Foxp3^+^ Treg cells (25). Interestingly, early restoration of circulating Treg numbers was predictive for treatment response at 3 months post-transplantation.

### Impact of Auto-HSCT for CD on TCR Repertoire Profile

The extensive immune renewal that follows auto-HSCT is associated with a vast diversification of the TCR repertoire. For example, in MS, CD4^+^ T cell dominant TCR clones that were present in peripheral blood before treatment were undetectable after immune reconstitution, with patients predominantly developing a new repertoire. More importantly, the T cell repertoire early during the reconstitution process were less diverse in patients who failed to respond to treatment (40). However, these data were exclusively obtained from peripheral blood and not from the site of inflammation. By contrast, next-generation sequencing (NGS) of the TCRβ locus in ileal and colonic biopsies collected at baseline (pre-mobilization) and after auto-HSCT (6 months and/or 1 year after transplantation) has been used to correlate TCR diversity with clinical and endoscopic outcome in 16 patients with CD (26). Quantifying repertoire similarities of T cell clones at different time points were quantified by the Morisita–Horn index (M–H; range 0–1) revealed monoclonal expansions in the mucosal T cell compartment at baseline prior to HSCT. The TCR repertoire was more polyclonal than expected (500 to 20,000 unique TCR sequences, clonality index 0.1 to 0.3), with no shared public TCR sequences being apparent in the mucosa of different patients. The clonality of the TCR in the mucosa was significantly increased after HSCT and the T cell repertoire appears reset, as the similarity index between baseline and after the procedure was low (26).

### Impact of G-CSF on Immune Reconstitution Post Auto-HSCT in CD

Granulocyte colony-stimulating factor is given both to mobilize hematopoietic stem cells and to accelerate neutrophil recovery post auto-HSCT. However, it can profoundly affect innate and adaptive immunity by inducing the differentiation of type 1 Treg cells and tolerogenic dendritic cells (DCs) (41). Clinical benefit from G-CSF was reported in five patients with severe endoscopic postoperative recurrence of CD who received 300 µg of recombinant human G-CSF three times per week for 12 consecutive weeks (42). Administration of G-CSF was safe and associated with significant increases in neutrophil counts, and levels of IL-1 receptor antagonist and soluble TNF receptor p55 and p75.

A clinical trial in nine patients with CD at the USA National Institutes of Health confirmed the potential benefit of subcutaneous G-CSF given over a 4-week period (43). In this study, changes in immune cell phenotypes, including myeloid and plasmacytoid DCs, as well as cytokine production were measured in the peripheral blood and colonic mucosal tissues. Patients who responded to G-CSF or achieved CR had significantly more IL-10-producing CD4^+^ memory T cells in the periphery, as well as a greatly enhanced CD123^+^ plasmacytoid DC infiltration of the lamina propria. Interestingly, IFN-γ production was higher in non-responders to G-CSF compared with responders. Although a randomized controlled placebo trial has not yet been performed, these studies suggest that G-CSF *per se* might offer some therapeutic benefit for patients with CD. Whether stem cell mobilization with G-CSF affects the functional orientation of immune cell populations in patients with CD and whether G-CSF-mobilized immune cell subsets, such as Treg cells and/or plasmacytoid DCs, favor the re-establishment of immune tolerance after auto-HSCT remains to be evaluated.

### Does Auto-HSCT Induce a Reset or Temporary Downregulation of Immunity in CD?

It is essential to determine whether there is a real “reset” of autoimmunity following auto-HSCT for CD, as has been observed for multiple sclerosis and systemic lupus erythematosus and which forms the basis for treatment-free remissions, or simply a temporary downregulation of innate and acquired immune responses. The relapse rate in CD after auto-HSCT is considerably higher compared to other ADs, which could be related to an insufficient eradication of the autoreactive immunologic memory by the conditioning regimens applied and/or a more prominent genetic contribution that favors disease recurrence. The latter fact is supported by epidemiologic studies demonstrating concordance rates among monozygotic twins of up to 50% (44). Alternatively, failure of achieving durable treatment-free remissions in CD post-transplantation could indicate that aberrant adaptive autoimmune responses and formation of a pathogenic immunologic memory are not the driving force in disease pathogenesis, as confirmed for other systemic ADs. In fact, perturbations in the innate immune pathway resulting in compromised mucosal barrier functions may have a stronger implication in driving chronic autoimmune responses in CD, which may not be corrected by “resetting” the immune system with auto-HSCT. Data supporting these considerations are not available yet, as studies investigating the influence of intestinal epithelial barrier changes on the dysfunction of mucosal innate and acquired immune responses after auto-HSCT in patients with CD are lacking.

### Future Directions for Immune Monitoring After Auto-HSCT in CD

Guidelines and expert recommendations to develop and implement systematic approaches to monitor immune responses in patients with cancer have been recently published (45). Likewise, the EBMT Autoimmune Diseases and Immunobiology Working Parties have initiated a joint process to develop and implement guidelines for “good laboratory practice” to provide practical recommendations for biobanking and immune monitoring in patients with ADs undergoing HSCT (46). The analysis of biological specimens at the cellular, DNA, transcriptional, epigenetic, posttranscriptional, and protein levels, including peripheral blood and tissues, yields a massive amount of data, which need to be processed with novel bioinformatics methods.

For the analysis of TCR repertoire, several different approaches and methods exist. The approach to be used is dependent on the experimental questions being asked and methodological bias can make it difficult to compare results across different studies. The latest advances, available tools, the choice of starting material, and the method for preparing samples have been reviewed in detail elsewhere (47). Most of the approaches involve the analysis of samples at the molecular level, commonly using deep and NGS (47).

Comprehensive protocols relating to multi-parameter flow cytometric analysis have been published previously (48, 49). In the future, such conventional flow cytometry may be combined with methods providing higher resolution, such as cytometry by time of flight mass spectrometry, as has already been used by Karnell and colleagues to investigate the kinetics of immune cell subset reconstitution in the periphery after HSCT and the impact of HSCT on the phenotype of circulating T cells in patients with MS (50). In addition, cytometric profiling may be complemented by an innovative flow cytometry approach which combines three monoclonal antibodies with two fluorophores to quantitate the TCR Vβ repertoire of human T lymphocytes (IOTest^®^ Beta Mark TCR V-beta Repertoire Kit, Beckman Coulter).

### Analysis of T Cell Reconstitution Based on T Cell Receptor Excision DNA Circles (TRECs)

T cell reconstitution after successful HSCT can occur *via* a thymic-independent pathway, which involves the expansion of graft-derived mature donor T cells, or a thymic-dependent pathway, the consequence of which is a regeneration of T cells with a more diverse TCR repertoire from graft-derived precursor cells (51). As thymic function is required for the *de novo* generation of T cells after transplantation, the potential function of T lymphopoiesis after auto-HSCT can be determined by quantifying TRECs (52). Signal joint TCR excision DNA circles (sjTRECs) result from the rearrangement of the TCR gene and the excision of circular DNA fragments from genomic DNA during thymocyte development. Measuring thymic function by quantifying sjTRECs in peripheral blood avoids disadvantages that are associated with the use of T cell surface molecules, such as CD45RA, as markers for recent thymic emigrants (RTEs). sjTRECs reflect developmental proximity to the thymus and the analysis of total sjTRECs levels and TCR beta variable region (TRBV) subfamily sjTRECs frequencies during immune reconstitution after HSCT is useful for more precisely determining thymic output function and T cell immune reconstitution (53). Although such analyses have not yet been undertaken in the context of CD, the increased precision of this approach has the potential to provide a more robust insight into the relationship(s) between immune status, disease status, and therapeutic resistance after auto-HSCT.

### Amplification-Free Gene Expression Profiling of the Periphery and Tissue

Gene expression profiles reflect the immune *milieu* and are increasingly being used for immune monitoring purposes and identifying predictive biomarkers in patients with cancer. Microarrays have traditionally supported the high-throughput analysis of gene and miRNA expression, but they are limited by the requirement for relatively large quantities of high-quality RNA. Next Generation Sequencing (NGS) has become an important discovery tool, and the preferred choice for unbiased biomarker discovery of transcriptional signatures associated with disease activity, treatment outcomes, and mechanism of action studies of therapeutic agents. However, data analysis and interpretation require advanced bioinformatics approaches. Quantitative PCR (qPCR) provides a more accurate insight into gene expression than microarrays and requires lower amounts of RNA. However, the analysis of the expression of multiple genes with classical qPCR and digital PCR, which can also be used for high-throughput high-precision analysis, is more difficult.

NanoString-based molecular “bar coding” enables the high-throughput analysis of the expression of multiple genes using defined panels such as the nCounter™ Human Immunology, Human Inflammation, and Human Myeloid Innate Immunity Panels. The Human Immunology module could be particularly relevant to the post-HSCT immune monitoring of patients with CD, given that the ASTIC trial reported treatment-related infections to be the most frequent serious adverse events during the 100 days after conditioning and the subsequent follow-up (17). The NanoString nCounter™ analysis system detects the expression of up to 800 genes in a single reaction with high sensitivity and linearity across a broad range of expression levels (54). The platform utilizes digital detection and direct molecular barcoding of individual target molecules using fluorescently labeled capture and reporter probes incorporating 35- to 50-base target-specific sequences. This technology allows for direct, PCR amplification-free multiplexed measurements of gene expression from a low amount of mRNA (25–300 ng). Different sources of RNA, including total RNA, fragmented RNA and formalin-fixed paraffin-embedded (FFPE)-derived RNA, can be used. This approach is suitable for the discovery of gene expression signatures, their validation and diagnostic testing in large translational studies.

### The Autologous Stem Cell Transplantation In Refractory CD—Low Intensity Therapy Evaluation (ASTIClite) TRIAL: An Opportunity to Investigate Immune Reconstitution Post Auto-HSCT in CD

Given that reduced intensity mobilization and conditioning regimens are associated with lower morbidity in malignant and AD (20, 55–58), the hypothesis that auto-HSCT using a reduced dose cyclophosphamide mobilization and low intensity conditioning (HSCTlite) will induce regression of ileocolonic ulceration in patients with refractory CD compared to standard of care will be tested *via* a soon-to-commence clinical trial (ASTIClite). This UK NIHR-funded national multicenter randomized controlled clinical trial aims to recruit 99 patients with a 2:1 randomization to compare HSCTlite with standard of care. For this, eligible outpatients with treatment refractory CD will be randomized to auto-HSCT with cyclophosphamide 1 g/m^2^ + G-CSF for mobilization followed by transplant conditioning with fludarabine 125 mg/m^2^, cyclophosphamide 120 mg/kg, and rabbit anti-thymocyte globulin 7.5 mg/kg (total doses) versus standard of care and followed for 48 weeks. The reduced doses of cyclophosphamide in the mobilization and conditioning regimens are based on the concerns raised following the single case of TRM in the original ASTIC trial, which may have been related to cumulative toxicity from higher mobilization dose of cyclophosphamide (4 g/m^2^) followed by the transplant conditioning (21, 22). It is hoped that this measure will improve overall safety and reduce the likelihood of neutropenic sepsis during the mobilization phase, which may be performed as an outpatient procedure.

The standard care group will be able to receive any licensed biologic, immunosuppressive or nutritional therapy for CD at the discretion of the treating physician. Pre-specified interim analyses will be undertaken to confirm that the mobilization regimen is effective for stem cell harvest with no negative impact on disease activity. Colonoscopy and MRI at week 24 will assess the requirement for re-initiation of maintenance of anti-TNF therapy in those with evidence of disease activity post auto-HSCT. Immunological profiling of blood and mucosa before and after HSCTlite will identify its mechanism of action. It is expected that HSCTlite will have an acceptable side-effect profile.

Additional secondary clinical endpoints will be the impact of HSCTlite on clinical disease activity, steroid requirements, quality of life, and the presence of adverse/serious adverse events, as compared to standard of care [and historical HSCT, as observed in ASTIC (16, 17)]. Exploratory endpoints will be the safety and efficacy of maintenance anti-TNF therapy in those patients with the recurrence of endoscopic disease after HSCTlite.

**Figure 2:**
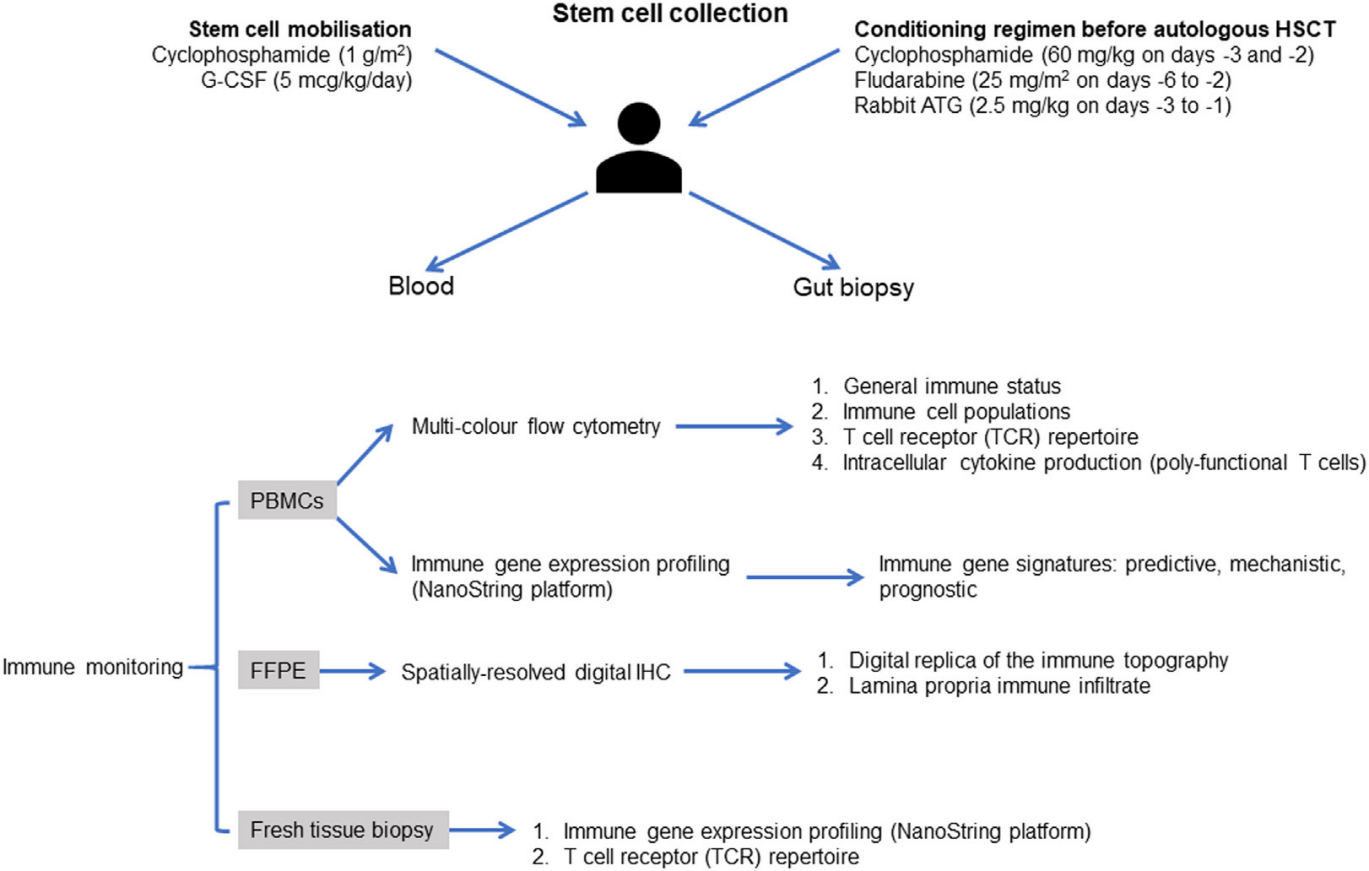
Proposed approach to immune monitoring in the Autologous Stem cell Transplantation In refractory CD—Low Intensity Therapy Evaluation clinical trial. Immune reconstitution will be evaluated using patient-derived blood mononuclear cells at weeks 8 12, 24, 32, and 48 after infusion autologous hematopoietic stem cells. Multi-color flow cytometry and optimized multicolor immunofluorescence-like antibody panels will be used to assess the general immune status of the patients, as well as the ability to release immune regulatory cytokines at the single-cell level. Mucosal biopsies will be assessed at baseline, weeks 24 and 48. RNA will be extracted and will be analyzed on a NanoString FLEX™ gene expression profiling platform, as detailed in the main text. Additional studies could employ a multiplexed spatial protein profiling approach based on NanoString digital quantification of tissue proteins to generate a digital replica of the immune topography of intestinal biopsies and to gain mechanistic insights into the immune determinants of response after autologous HSCT, with emphasis on the reconstitution of plasmacytoid DCs, regulatory T cells, and TCR-Vβ repertoires. Abbreviations: G-CSF, granulocyte colony-stimulating factor; Cy, cyclophosphamide; Flu, fludarabine; PBMCs, peripheral blood mononuclear cells; FFPE, formalin-fixed paraffin-embedded; IHC, immunohistochemistry.

At the core of this study is comprehensive and informative immunological profiling of the periphery and mucosa before and after treatment (Figure 2). Profiling will be focused on generating insight into the pathogenesis of CD, the responsiveness, and restoration of responsiveness to anti-TNF therapy in patients with endoscopic disease recurrence and the immunological events that precede recurrence of disease and occurrence of resistance to anti-TNF therapy in patients that relapse after auto-HSCT.

Reconstitution of immune potential is being monitored by determining the re-appearance of monocyte subsets, T cell subsets (“helper,” “cytotoxic,” naïve, central memory, effector memory, regulatory, RTEs), B cell subsets, and NK cell subsets (cytokine-secreting CD3^−^CD56^bright^CD16^+/−^ and cytotoxic; CD3^−^CD56^dim^CD16^+^) using multi-parameter flow cytometry. Representative panels that can be used for the monitoring of immune reconstitution in patients with CD following auto-HSCT are summarized in Table 1, the ability to deliver which will be dependent on the instrumentation available in the analytical laboratory. Comprehensive protocols relating to flow cytometric analysis have been published previously (48, 49).

**Table 1:** Flow cytometry antibody panels for immune cell reconstitution in CD.

Cell type/subtype	Phenotype
**CD4^+^ and CD8^+^ T cells^a^**	
Naïve	CD3^+^CD4^+/−^CD8^−/+^CD45RA^+^CCR7^+^
Effector	CD3^+^CD4^+/−^CD8^−/+^CD45RA^+^CCR7^−^
Central memory	CD3^+^CD4^+/−^CD8^−/+^CD45RA^−^CCR7^+^
Effector memory	CD3^+^CD4^+/−^CD8^−/+^CD45RA^−^CCR7^−^
Gut-homing T cells	CD3^+^CD4^+/−^CD8^−/+^CD49d^+^α4integrin^+^CCR9^+^
Recent thymic emigrants	CD3^+^CD4^+/−^CD8^−/+^CD31^+^

**CD4^+^ T helper (Th) subsets (option to include CD45RA^+/−^)^a^**	
Th1	CD3^+^CD4^+^CD8^−^CXCR5^−^CXCR3^+^
Th2	CD3^+^CD4^+^CD8^−^CXCR5^−^CCR4^+^CCR6^−^
Th9	CD3^+^CD4^+^CD8^−^CXCR5^−^CCR4^−^CCR6^+^
Th17	CD3^+^CD4^+^CD8^−^CXCR5^−^CCR4^+^CCR6^+^CCR10^−^
Th17.1	CD3^+^CD4^+^CD8^−^CXCR5^−^CXCR3^+^CCR6^+^CCR4
Th22	CD3^+^CD4^+^CD8^−^CXCR5^−^CCR4^+^CCR6^+^CCR10^+^
Follicular T helper (Tfh)	CD3^+^CD4^+^CD8^−^CXCR5^+^PD-1^+^ICOS^+^
Gamma delta T cells	CD3^+^TCRγδ^+^

**CD4^+^ immunoregulatory T (Treg) cells**	
	CD3^+^CD4^+^CD25^high^CD127^low^Foxp3^+^CCR4^+/−^CD45RO^+/−^

**Myeloid-derived suppressor cells (MDSCs)—Lineage (Lin) cocktail: CD3/CD19/CD20/CD56**	
Monocytic	Lin^−^HLA-DR^−/low^CD11b^+^CD14^+^CD15^−^CD124^+^
Granulocytic	Lin^−^HLA-DR^−/low^CD11b^+^CD14^−^CD15^+^CD124^+^
Endothelial progenitor	Lin^−^HLA-DR^−/low^CD11b^+^CD124^−^

**Dendritic cells (DCs)—lineage (Lin) cocktail: CD3/CD19/CD20/CD56**	
Plasmacytoid	Lin^−^CD14^−^CD123^+^CD11c^−^
Conventional	Lin^−^CD14^−^CD123^−^CD11c^+^

**Innate lymphoid cells (ILCs)—lineage (Lin) cocktail: CD3/CD14/CD19/CD20**	
ILC1	Lin^−^CD127^+^CD161^+^CD117^−^CD294^−^NKp44^−^
ILC2	Lin^−^CD127^+^CD161^+^CD294^+^
ILC3	Lin^−^CD127^+^CD161^+^CD117^+^CD294^−^NKp44^−^

**Mast cell progenitors (MCPs)—Lineage (Lin) cocktail: CD3/CD14/CD19/CD20**	
	Lin^−^FcεRIα^+^CD203^+^CD117^+^

**Natural killer (NK) cell subsets**	
Cytotoxic	CD3^−^CD56^dim^CD16^+^
Cytokine-producing	CD3^−^CD56^bright^CD16^+/−^

**Monocytes**	
Classical	CD3^−^CD14^high^CD16^-^
Intermediate	CD3^−^CD14^high^CD16^+^
Non-classical	CD3^−^CD14^+^CD16^high^

**B cells/plasmablasts**	
Naïve	CD3^−^CD19^+^CD27^−^IgD^+^
Switched memory	CD3^−^CD19^+^CD27^+^IgD^−^
Non-switched memory	CD3^−^CD19^+^CD27^+^IgD^+^
Plasmablasts	CD3^−^CD19^+^CD27^high^IgD^−^CD38^high^
Regulatory B cells	CD3^−^CD19^+^CD1d^high^CD5^+^CD21^+^CD24^high^

**T cell responsiveness to stimulation**	
	CD3^+^CD4^+^CD8^−^IL2^−/+^IL4^−/+^IL17^−/+^TNFα^−/+^IL10^−/+^IFNγ^−/+^
	CD3^+^CD4^−^CD8^+^IL2^−/+^IL4^−/+^IL17^−/+^TNFα^−/+^IL10^−/+^IFNγ^−/+^

*^a^CD4 differentiation and helper subsets can be gated upon after Foxp3^+^CD25^hi^CD127^low^ Treg cells have been excluded from the CD4^+^ T cell parent population*.

Combined with post-transplant vaccination, the immune reconstitution of “ASTIClite” will therefore aim to establish whether the effect of AHSCT is merely temporary downregulation of immunity or whether there is a significant component of immune reset.

## Conclusion

Based on clinical trials and EBMT registry data, auto-HSCT represents a promising therapy for patients with severe resistant CD. Prolonged responses have been achieved in some patients that have otherwise been resistant to conventional treatments and biological therapies. In the patients who relapse, there appears to be a re-sensitization to previous agents, consistent with a “setting back of the immunological clock.” Previous clinical trials and case series report a relatively high relapse rate and high frequency of serious adverse events. Future trials will assess the efficacy and safety of lower intensity mobilization and conditioning regimens and the benefit of protocoled introduction of maintenance therapy in patients who relapse after auto-HSCT.

In addition, clinical trials of auto-HSCT provide a unique opportunity to characterize the nature of immune reconstitution as well as the interaction between the peripheral and mucosal immune system in CD. This will allow deep interrogation and characterization of the localized mucosal immune environment in patients with disease before and after auto-HSCT during ASTIClite, as well as the immunome of the periphery (by profiling peripheral blood mononuclear cells, PBMCs) before and after auto-HSCT and during the progression toward disease relapse.

Recently, the EBMT and European Crohn’s and Colitis Organisation have published a review to encourage and guide inter-specialty collaboration in both clinical and scientific development of this auto-HSCT in CD (59). Thus, in addition to therapeutic benefits, destroying and re-building the dysfunctional immune system and mucosal environment, a program of modern scientific investigation carefully scheduled around auto-HSCT may yield valuable insights into the etiology, pathogenesis, and mechanisms of treatment resistance in CD.

## Author Contributions

AP drafted the initial version of the manuscript, to which all other authors made significant content and editorial contributions, with JS and TA leading the contribution from the EBMT Autoimmune Diseases Working Party. All authors are integrally involved in the clinical and/or scientific aspects of the ASTIClite trial (Chief Investigator, JL).

## Disclaimer

Although drug doses have been checked against primary sources, this review should not be used as a means of assuring prescription of chemotherapy and other drugs in clinical practice.

## Conflict of Interest Statement

JS has received honoraria for speaking from Sanofi and Jazz. JG has received honoraria for advisory boards from Abbvie, Celgene, Gilead, Janssen, Roche/Genentech, and Novartis. Otherwise, the authors confirm that there are no commercial or financial interests and relationships that could be construed as being a potential conflict of interest. The handling Editor declared a past co-authorship with one of the authors JS, and a shared affiliation with the reviewer MR.
